# Exposure to dim light at night prior to conception attenuates offspring innate immune responses

**DOI:** 10.1371/journal.pone.0231140

**Published:** 2020-04-17

**Authors:** Yasmine M. Cissé, Kathryn Russart, Randy J. Nelson

**Affiliations:** 1 Department of Pharmacology, University of Maryland School of Medicine, Baltimore, Maryland, United States of America; 2 Department of Neuroscience, The Ohio State University Wexner Medical Center, Columbus, Ohio, United States of America; 3 Department of Neuroscience, Rockefeller Neuroscience Institute, West Virginia University, Morgantown, West Virginia, United States of America; University of Lübeck, GERMANY

## Abstract

Functional circadian timekeeping is necessary for homeostatic control of the immune system and appropriate immune responsiveness. Disruption of natural light-dark cycles, through light at night (LAN), impairs innate and adaptive immune responses in nocturnal rodents. These altered immune responses are associated with disrupted endogenous gene transcriptional and endocrine cycles. However, few studies have addressed the multigenerational consequences of systemic circadian rhythm disruption. We hypothesized that parental exposure to dim LAN (dLAN) would alter innate immune and sickness responses to an endotoxin challenge in adult offspring gestated and reared in dark nights. Adult male and female Siberian hamsters were exposed to either dark nights (DARK) or dLAN (~5 lux) for 8 weeks, then paired, mated, and thereafter housed under dark nights. Maternal exposure to dLAN prior to conception impaired febrile responses and increased splenic *il-1* production in response to LPS in male offspring. Paternal pre-conception dLAN dampened offspring *tnf*-**α** expression in the hypothalamus, reduced serum bactericidal capacity, and dark phase locomotor activity. These changes occurred despite offspring being conceived, gestated, and reared under standard dark night conditions. Overall, these data suggest that dLAN has intergenerational effects on innate immunity and sickness responses.

## Introduction

The endogenous circadian system provides temporal organization of competing energetic and physiological processes to optimize survival and reproduction. One such circadian-regulated process is immune function. Virtually all immune cells and tissues possess intrinsic clocks. Clock genes have been implicated in direct and indirect regulation of immune cell survival and inflammatory responses. CLOCK protein up-regulates transcription of NF-κB-responsive genes during the inactive phase, whereas BMAL1 and CRY proteins limit NF-κB downstream transcription and activation during the active phase, respectively [1,2]. Additionally, REV-ERBα regulates circadian gating of proinflammatory cytokine production in macrophages [3]. Proper circadian timekeeping is necessary for homeostatic control of the immune system and appropriate immune responsiveness.

As suggested, elimination of a functional circadian clock through clock gene deletion or ablation of the central circadian pacemaker, the suprachiasmatic nucleus (SCN), results in aberrant circadian gating of immune function and responsiveness. Disruption of timekeeping mechanisms at the level of the SCN diminishes febrile responses, delays wound healing, and eliminates rhythms in various immune processes [4–6]. Disruption of natural light/dark cycles, through exposure to light at night (LAN), impairs innate and adaptive immune responses in nocturnal rodents [7–9]. These altered immune responses are associated with disruption of endogenous cycles in transcription, hormone production, and homeostatic processes. However, few studies have addressed the multigenerational consequences of systemic circadian disruption.

Our laboratory has previously reported that parental exposure to dim LAN (dLAN) impairs offspring adaptive immunity and decreases global splenic methylation [10]. The spleen is central to the propagation of an inflammatory signal and altered splenic methylation may underlie differential responses to innate immune challenges. We predicted that parental exposure to dLAN would decrease febrile, central and peripheral cytokine responses to an endotoxin challenge in adult offspring gestated and reared in dark nights.

## Methods

### Animals

Male (n = 19) and female (n = 19) Siberian hamsters (*Phodopus sungorus*) were obtained from our in-house breeding colony at The Ohio State University. Hamsters were maintained on a standard 16h-8h light-dark cycle (DARK; 125:0 lux or 33.2:0 mW/cm^2^ at 550 nm, in accordance with [11] reporting) with lights illuminated at 22.00 h and terminated at 14.00 h EST, in polypropylene cages (30 x 15 x 14cm) on static racks in a temperature- and humidity-controlled vivarium. All animals were provided *ad libitum* access to food (Harlan Teklad 7912; Madison, WI, USA) and filtered tap water. All experiments were approved by the Ohio State University Institutional Animals Care and Use Committee (Protocol 2008A0162), and animals were maintained in accordance with the recommendations of the National Institutes of Health and *The Guide for the Care and Use of Laboratory Animals*.

#### Generation of F1 animals

Adult (four to six months of age) male and female Siberian hamsters were individually housed and randomly assigned to an experimental lighting condition comprising dark nights (DARK) or exposed to dLAN (125:5 lux or 33.2:1.8 mW/cm^2^). dLAN was supplied using LUMA5 LED light strips (1.5 W/ft, 5000 K “cool white”, 1200 lumens: Hitlights Inc.; Louisiana, USA) placed parallel and equidistant from cage level. Light levels were measured in an empty cage, from the center, using a Malvolux 5032C illuminance meter (Nürnberg, Germany) and an Ophir Starbright irradiance meter (Jerusalem, Israel) with light sensors facing upward. Measurements were recorded within one year of both devices being purchased, therefore meters had not yet needed recalibration. Cage positions on rack were changed weekly, during cage changes, to account for possible positional effects. Hamsters were maintained in their respective lighting conditions for 8 weeks at which point all animals were paired, mated, and thereafter housed in standard dark night conditions. Pairings resulted in four parental groups with the following night conditions: Dark-Dark (Male/Female), Dark-dLAN, dLAN-Dark, and dLAN-dLAN. Males were removed one week after pairing. Pups were weaned at 21 days of age and group-housed with same sex siblings. Experiment 1 was conducted with a total of 46 males: Dark-Dark (n = 6 saline vs n = 4 LPS), Dark-dLAN (n = 6 saline vs n = 6 LPS), dLAN-Dark (n = 6 saline vs n = 4 LPS), dLAN-dLAN (n = 7 saline vs n = 7 LPS). Experiment 1 was conducted with a total of 46 females: Dark-Dark (n = 6 saline vs n = 5 LPS), Dark-dLAN (n = 3 saline vs n = 5 LPS), dLAN-Dark (n = 7 saline vs n = 7 LPS), dLAN-dLAN (n = 6 saline vs n = 7 LPS). Experiment 2 was conducted with a total of 32 males: Dark-Dark (n = 4 saline vs n = 4 LPS), Dark-dLAN (n = 3 saline vs n = 4 LPS), dLAN-Dark (n = 4 saline vs n = 4 LPS), dLAN-dLAN (n = 5 saline vs n = 4 LPS). Experiment 2 was conducted with a total of 28 females: Dark-Dark (n = 4 saline vs n = 4 LPS), Dark-dLAN (n = 3 saline vs n = 4 LPS), dLAN-Dark (n = 3 saline vs n = 4 LPS), dLAN-dLAN (n = 3 saline vs n = 3 LPS). Offspring were randomly assigned to saline or LPS treatment groups.

### Experiment 1: Effect of parental dLAN on offspring febrile responses

#### Surgical procedures

Male and female offspring Siberian hamsters were implanted intraperitoneally (i.p.) with radio-telemetric transmitters (Mini-Mitter, Bend, OR) under isoflurane anesthesia and given one week to recover. Cages were then placed on TR-3000 receiver boards and connected to DP-24 DataPorts (Mini-Mitter) and a personal computer. Emitted temperature frequencies were collected in 10 min intervals (bins) and converted to temperature values by interpolating from programmed calibration curves of individual transmitters.

#### Fever assessment

Body temperature and locomotor activity were monitored for 5 days in order to establish a baseline prior to lipopolysaccharide (LPS) treatment. On Day 5, a subset of hamsters in each group received 0.1 cc i.p. injections of 0.5 mg/kg LPS (serotype 0111:B4; Sigma-Aldrich, St. Louis, MO) suspended in sterile saline prior to the onset of the active phase (ZT 15.5). The remaining hamsters received injections of saline. Changes in body weight, *ad libitum* food intake, body temperature, and activity were evaluated over 18 hours post-LPS.

### Experiment 2: Effect of parental dLAN on offspring innate immune responses

#### LPS administration

A second subset of adult male and female offspring received 0.1 cc i.p. injections of 0.5 mg/kg LPS prior to the onset of the active phase (ZT 15.5–16). Three hours post-LPS treatment (ZT 18.5–19), hamsters were lightly anesthetized with isoflurane vapors, and rapidly decapitated in order to collect blood and tissues. Blood samples were centrifuged for serum at 4°C for 30 min at 3500 rpm and stored at -80°C for future assessment of bactericidal capacity. Brains were removed and stored in RNALater reagent (Qiagen) for later dissection. Spleens were removed, weighed, and flash frozen on dry ice for subsequent qPCR analysis.

#### Blood bactericidal capacity

Under a laminar flow hood, plasma samples were diluted 1 : 20 in CO_2_-independent media (Gibco, Carlsbad, CA, USA). A standard number of colony-forming units (CFUs) of *Escherichia coli* (Epower 0483E7, Fisher Scientific) were added to each sample in a ratio of 1:10. Plasma-bacteria mixtures were then incubated for 30 min at 37°C and plated in duplicate onto tryptic-soy agar plates using a sterile technique. Two plates were spread with diluted bacteria alone as positive controls, and two were spread with media alone as negative controls. All plates were incubated at 37°C overnight, and total CFUs were quantified by an experimenter unaware of lighting conditions. Positive control plates yielded an average of 700 CFUs, whereas negative control plates did not grow any colonies. Total CFUs were averaged across the duplicates for each animal and then compared with the average of the positive control plates to calculate the percent of bacteria killed.

#### Quantitative PCR (qPCR)

Total RNA from spleens and hypothalami were extracted using RNAzol reagent (Sigma Aldrich, St. Louis, MO). RNA quality was assessed using a Nanodrop One Spectrophotometer (Thermo Scientific) and normalized to 200 ng/μL prior to reverse transcription into cDNA using M-MLV Reverse Transcriptase enzyme (Invitrogen, Carlsbad, CA) according to the manufacturer’s instructions. Primer sequences are as follows, *18S* forward, 5′-AGAAACGGCTACCACATCCAA-3’, *18S* reverse 5′-GGGTCGGGAGTGGGTAATTT-3′; *IL-1*β forward 5′- AAGATACCTGTGGCCTTGGGC -3′, *IL-1β* reverse 5′- AGGGTGGGTGTGTCACCTTT -3′; *Tnfα* forward 5′- GCAGATGGGCTGTACCTCGT-3′, *Tnfα* reverse 5′- GTGAGGAGCACGTAGTCGGA -3 ′; *IL-6* forward 5′- ACTGCCCTCCCTACCTCACA -3′, *IL-6* reverse 5′- TGGTGTGTACTGGTCTGTTGGG -3 ′. IL-1, IL6, and TNF gene expression was assessed on an ABI 7500 Fast Real Time PCR system using SYBR Green master mix (Invitrogen, Carlsbad, CA). Cycling conditions were: 95°C for 5 min, followed by 40 cycles of 95°C for 15 sec, and 60°C for 1 min. Any samples containing phenol contamination or less than 200 ng/μL of RNA were noted and eliminated from analysis if 18s rRNA did not amplify before cycle 14. Relative gene expression was calculated using the Pfaffl method [12]. Data are expressed as fold change from DARK-DARK saline-injected animals.

### Maternal care

Maternal care was observed and scored twice daily from postpartum day (PD) 2–8. Dams (Dark-Dark, n = 9; Dark-dLAN, n = 6; dLAN-Dark, n = 9; dLAN-dLAN, n = 9) were observed for 10 minutes during the middle of the light phase (ZT8-9) and prior to the onset of the dark phase (ZT13-14). During the observation period, dams were scored for engaging in the following behaviors: licking/ grooming, nursing and licking, nursing (passive, blanket, and arched back nursing), self-grooming, and time off-nest. Time spent engaging in each behavior was summated across observations. Fragmentation of maternal care was defined as number of interrupted bouts of nursing behavior during an observation.

### Data analyses

Baseline body temperatures for the active and inactive phases of the light cycle were determined for each animal using mean values for the 5 days prior to LPS. Temperatures were analyzed for the 18 h following LPS injections using a repeated measures ANOVA assessing the effects of parental lighting condition, offspring sex, and interactions. Fever was operationally defined as body temperatures significantly (p < 0.05) higher than the active phase baseline for each 10 min interval using two-tailed *t*-tests. The total duration of temperatures above the baseline, maternal care, gene expression data, and serum bactericidal capacity and were compared between groups using two-way ANOVAs. Animals were excluded from Experiment 1 if no significant circadian rhythm in baseline locomotor activity or body temperature was observed by Cosinor analysis. Samples were excluded from gene expression analysis if 18S amplified later than CT 13. Post-hoc differences between all means were analyzed using Tukey’s honestly significant difference (HSD) test. If the data did not meet the assumptions of normality or equal variance, then nonparametric statistical tests were conducted. Differences between means were considered statistically significant when *p* ≤ 0.05 for all analyses.

## Results

### Experiment 1

#### Male offspring of sires exposed to preconception dLAN reduced dark phase locomotor activity

**Fig 1:**
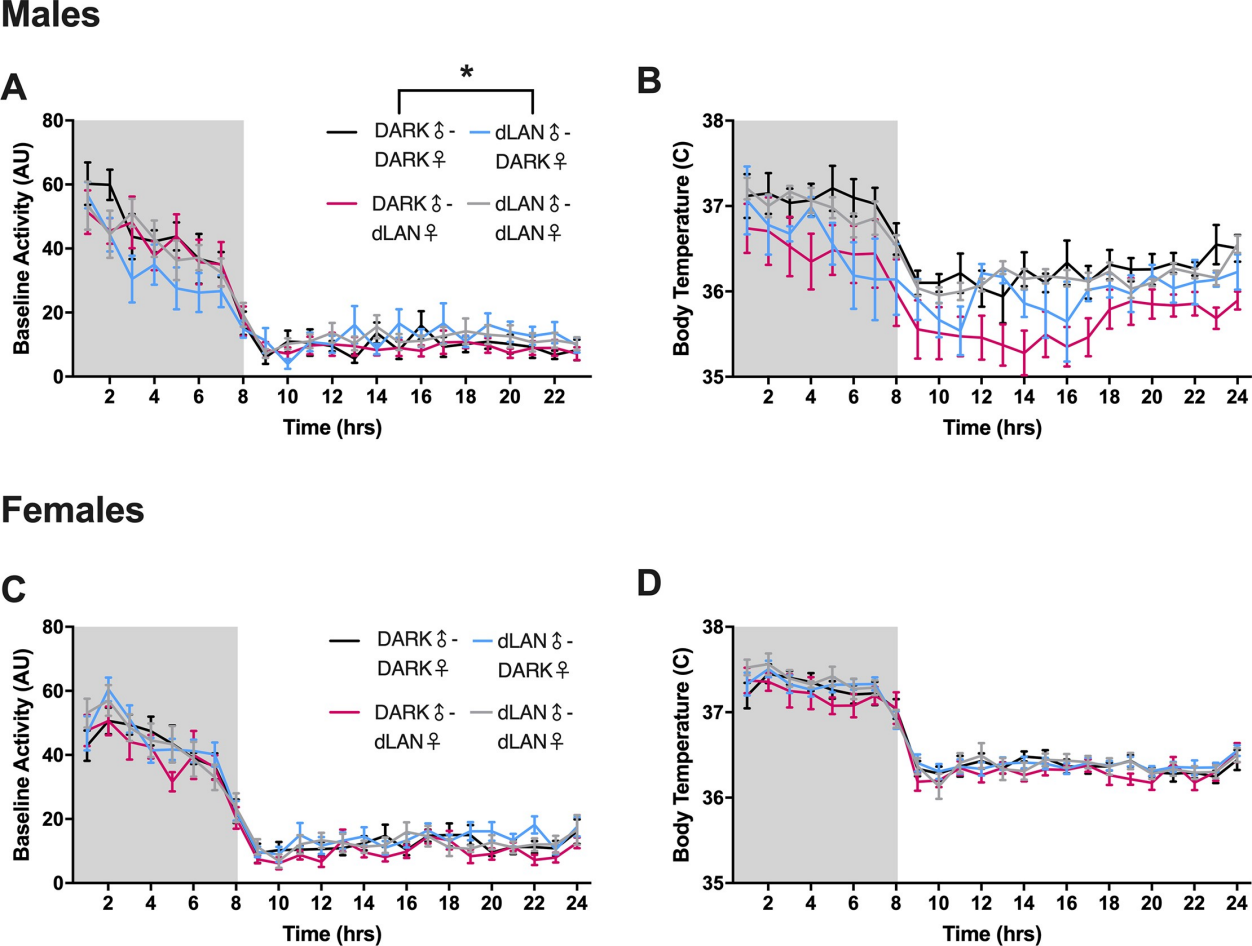
Male offspring of sires exposed to dLAN dampened locomotor activity rhythm amplitude. Homecage locomotor activity and body temperature rhythms in male (A,B), and female (C,D) offspring. N = 4-7/ group; error bars represent SEM. *, *P* < 0.05 Dark-Dark vs dLAN-Dark.

Male offspring of sires exposed to dLAN decreased locomotor activity rhythm amplitude relative to males of DARK-DARK parents (F_1,11_ = 14.49, *p* < 0.01: Fig 1A). Whereas male offspring of dams exposed to dLAN decreased average body temperature relative to males of DARK-DARK parents (F_3,12_ = 4.17, *p <* 0.05: Fig 1B). There was a sex difference in body temperature, such that females increased body temperature relative to males (F_47,445_ = 2.88, *p <* 0.05), but parental lighting did not alter overall locomotor or body temperature rhythms (Fig 1C and 1D).

#### Male offspring of dams exposed to preconception dLAN shortened fever duration

**Fig 2:**
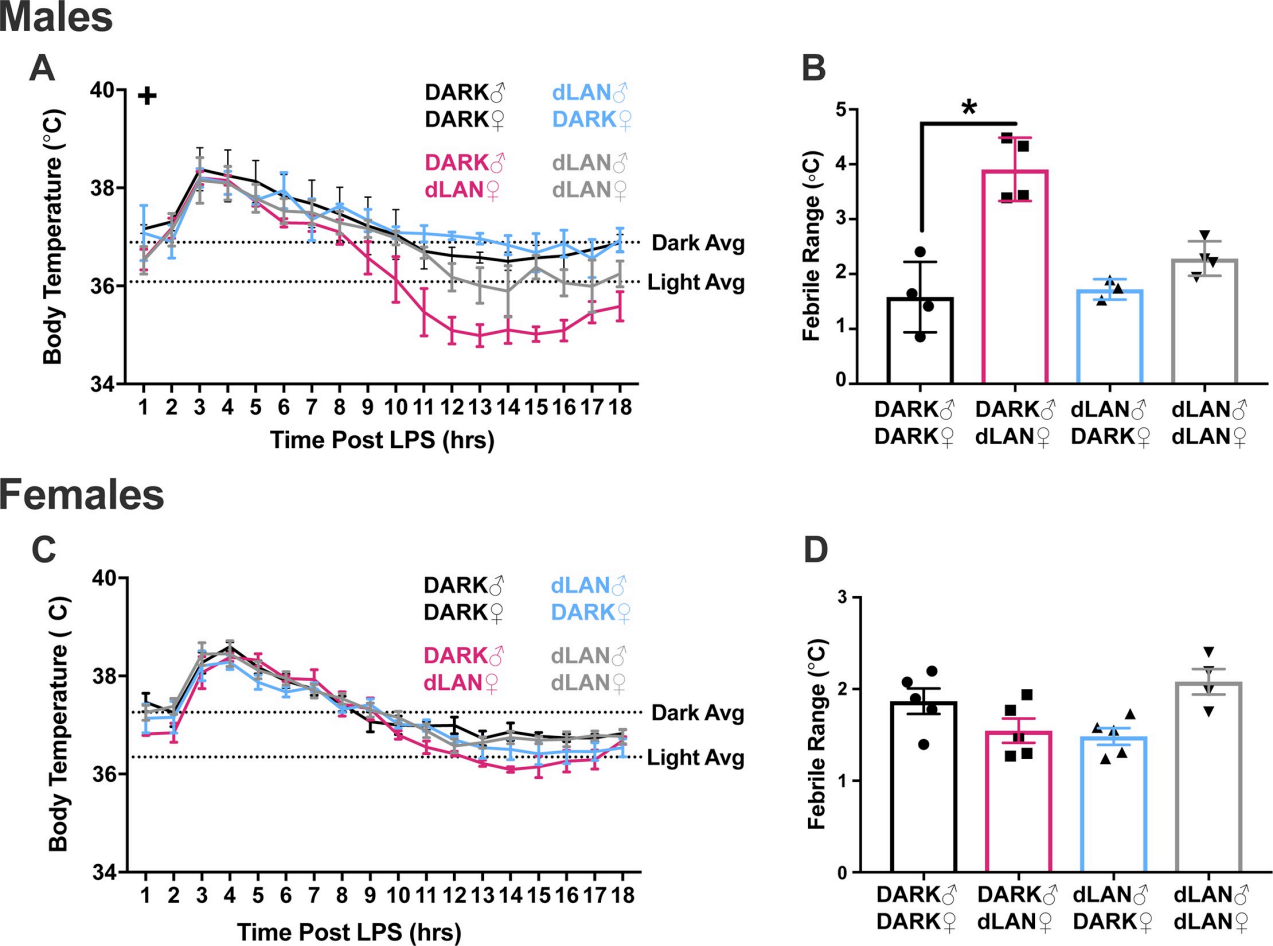
Male offspring of dams exposed to preconception dLAN enhanced magnitude of febrile responses. Body temperature from 0 to 18 hours post-LPS and febrile range in male (A,B) and female (C,D) offspring. Horizontal dashed lines represent mean baseline body temperature during the dark and light phase. N = 3-5/group/sex; error bars represent SEM. +, *P* < 0.05 Dark-dLAN vs Dark-Dark and dLAN-dLAN; *, *P* < 0.05 Dark-dLAN vs Dark-Dark.

Due to baseline and LPS-induced (F_6,147_ = 4.19, *p* < 0.05) sex differences in body temperatures, male and female febrile responses were analyzed separately. Male offspring of dams exposed to dLAN markedly reduced febrile response (F_3,8_ = 6.23, *p* < 0.05) relative to DARK-DARK and dLAN-dLAN male offspring (Tukey’s, *p* < 0.05; Fig 2A). However, the change in body temperature relative to baseline was enhanced in DARK-dLAN males relative to control males (F_1,9_ = 14.51, *p* < 0.05; Fig 2B).

LPS induced febrile response in females (F_17,153_ = 50.05, *p* < 0.001), but febrile responses were unaltered by parental exposure to dLAN (Fig 2C and 2D).

### Experiment 2

#### Preconception dLAN altered offspring LPS-induced splenic cytokine gene expression

**Fig 3:**
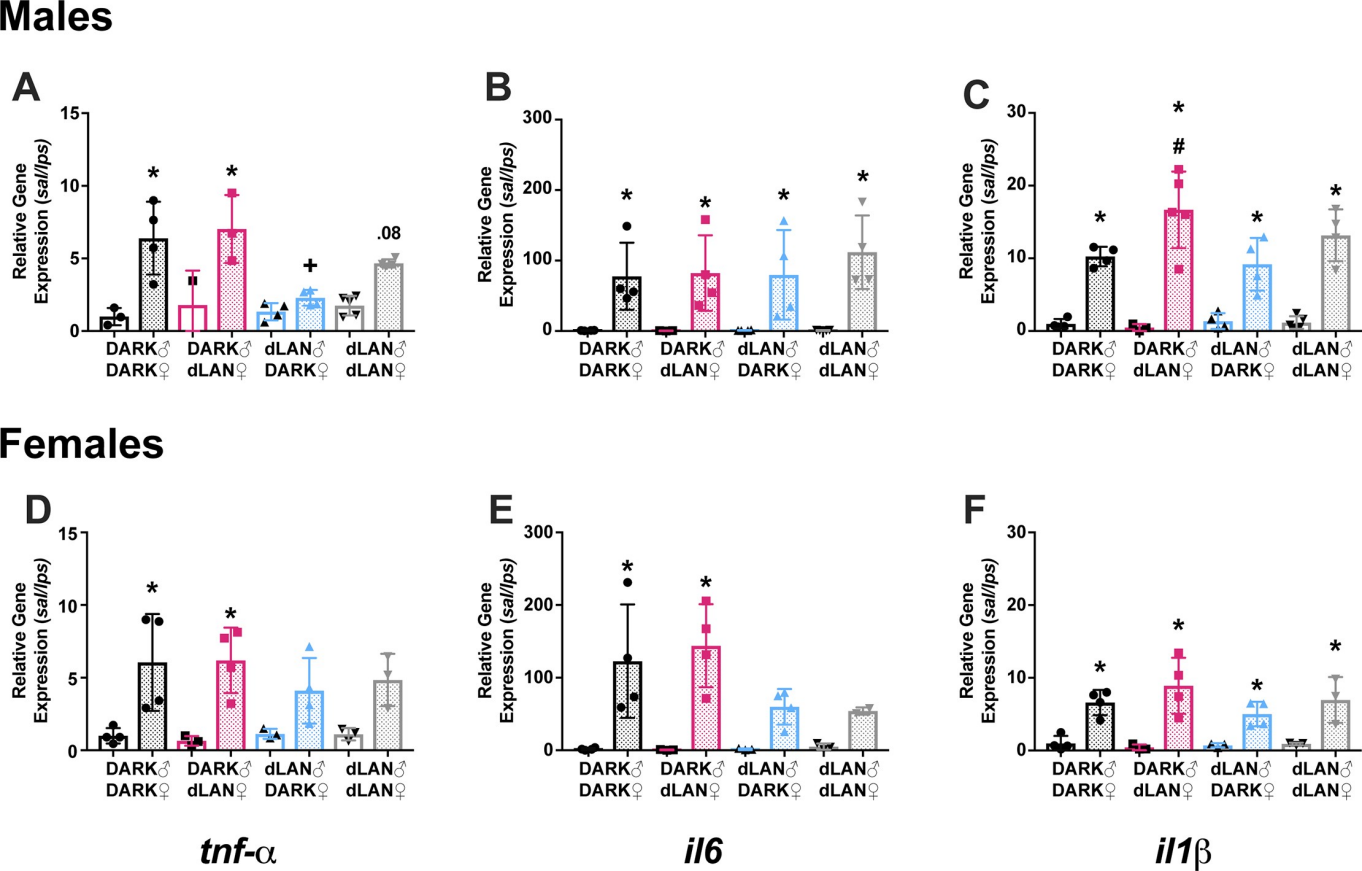
Preconception dLAN altered splenic cytokine gene expression in an offspring sex-specific manner. Splenic *tnf-α*, *il-6*, and *il-1* expression 3 hours post-LPS challenge in males (A-C) and females (D-F). Open bars represent saline, dotter bars represent LPS. N = 3-5/group/sex; error bars represent SD. #, *P* < 0.05 Dark-dLAN LPS vs Dark-Dark LPS and dLAN-Dark LPS; +, *P* < 0.05 dLAN-Dark LPS vs Dark-Dark LPS and Dark-dLAN LPS; *, *P* < 0.05 saline vs LPS.

Male offspring of sires exposed to dLAN decreased LPS-induced *tnf-α* gene expression in the spleen (F_3,21_ = 4.09, *p* < 0.05; Fig 3A), such that dLAN-Dark male LPS response was significantly dampened relative to control males (Tukey’s, *p* < 0.05). Male offspring of dLAN-exposed dams increased splenic *il-1* gene expression in response to LPS (F_3,25_ = 3.25, *p <* 0.05; Fig 3C), such that Dark-dLAN male offspring *il1* expression significantly increased relative to all other groups (Tukey’s, *p* < 0.05). Female offspring of dLAN sires dampened splenic *tnf-α* and *il-6* expression in response to LPS (F_1,20_ = 35.59 and F_1,19_ = 35.13, *p* < 0.0001, respectively; Fig 3D and 3E), such that dLAN-Dark and dLAN-dLAN females did not significantly increase cytokine gene expression relative to their saline-treated counterparts (Tukey’s, *p* < 0.05).

#### Preconception dLAN altered offspring LPS-induced hypothalamic cytokine gene expression

**Fig 4:**
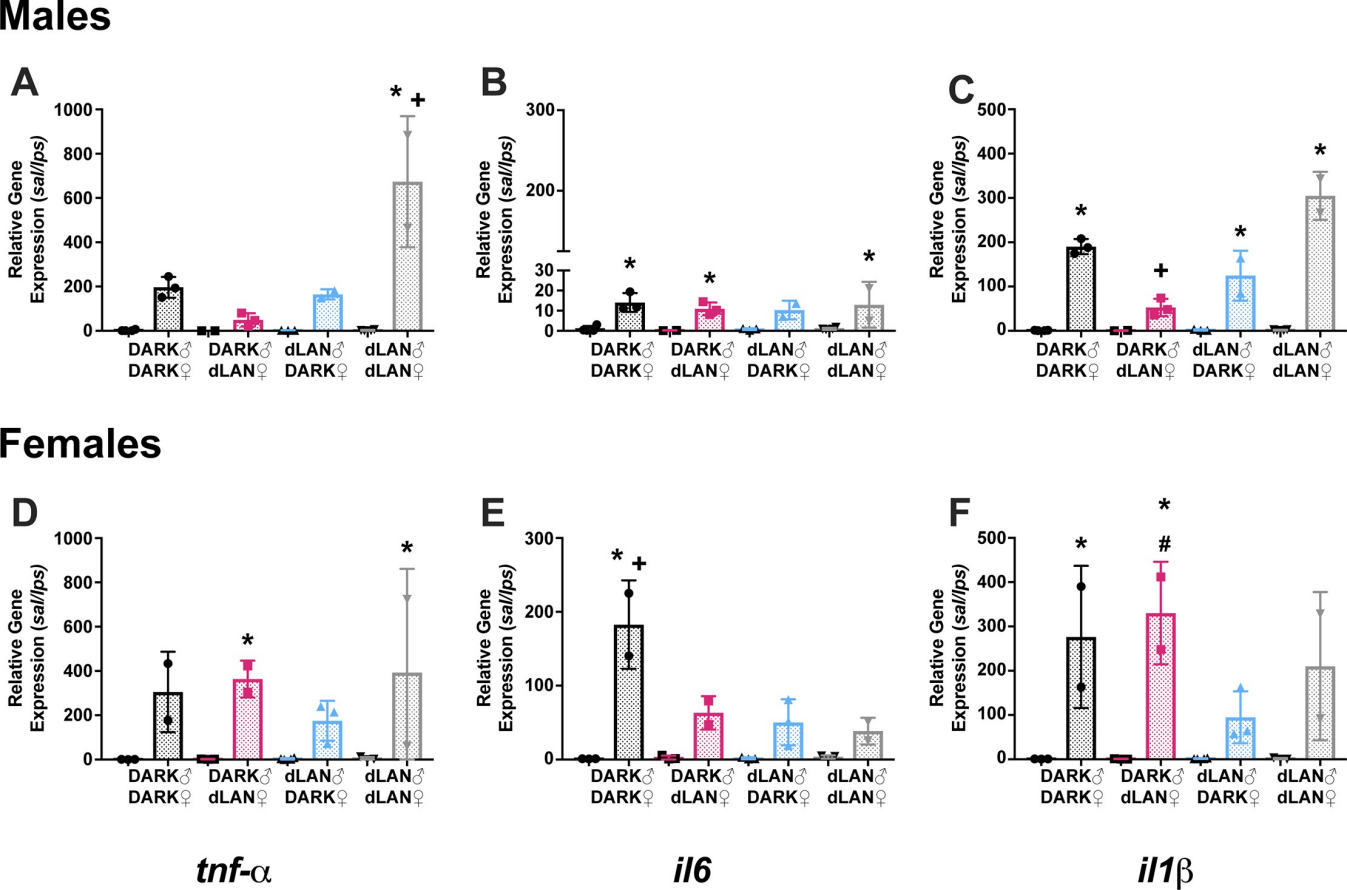
Paternal dLAN blunted hypothalamic cytokine gene expression in female offspring. Hypothalamic *tnf-α*, *il-1*, and *il-6* expression 3 hours post-LPS challenge in males (A-C) and females (D-F). Open bars represent saline, dotter bars represent LPS. N = 2-4/group/sex; error bars represent SD. +, *P <* 0.05 vs all LPS; #, *P <* 0.05 vs dLAN-Dark LPS; *, *P* < 0.05 saline vs LPS.

Male offspring of dams exposed to dLAN dampened male hypothalamic *il-1* expression (F_3,15_ = 15.72, *p < 0*.*05*; Fig 4C), such that Dark-dLAN male offspring significantly decreased LPS-induced *il-1* gene expression relative to all other groups (Tukey’s, *p* < 0.05). However, female offspring of sires exposed to dLAN dampened hypothalamic *il-1* response (F_1,14_ = 50.37, *p <* 0.05), such that *il-*1 expression was not significantly increased in LPS-injected relative to their saline treated counterparts (Tukey’s, *p* < 0.05). dLAN-dLAN male offspring increased LPS-induced expression of *tnf-α* in the hypothalamus relative to all other males (F_3,15_ = 15.72, *p < 0*.*05*; Fig 4A). Female offspring of parents exposed to dLAN dampened *il-6* response to LPS (F_3,13_ = 11.34, *p <* 0.001; Fig 4E), such that all groups decreased LPS-induced *il-6* gene expression relative to Dark-Dark females (Tukey’s, *p* < 0.05).

#### Preconception exposure to dLAN dampened offspring serum bactericidal capacity

**Fig 5:**
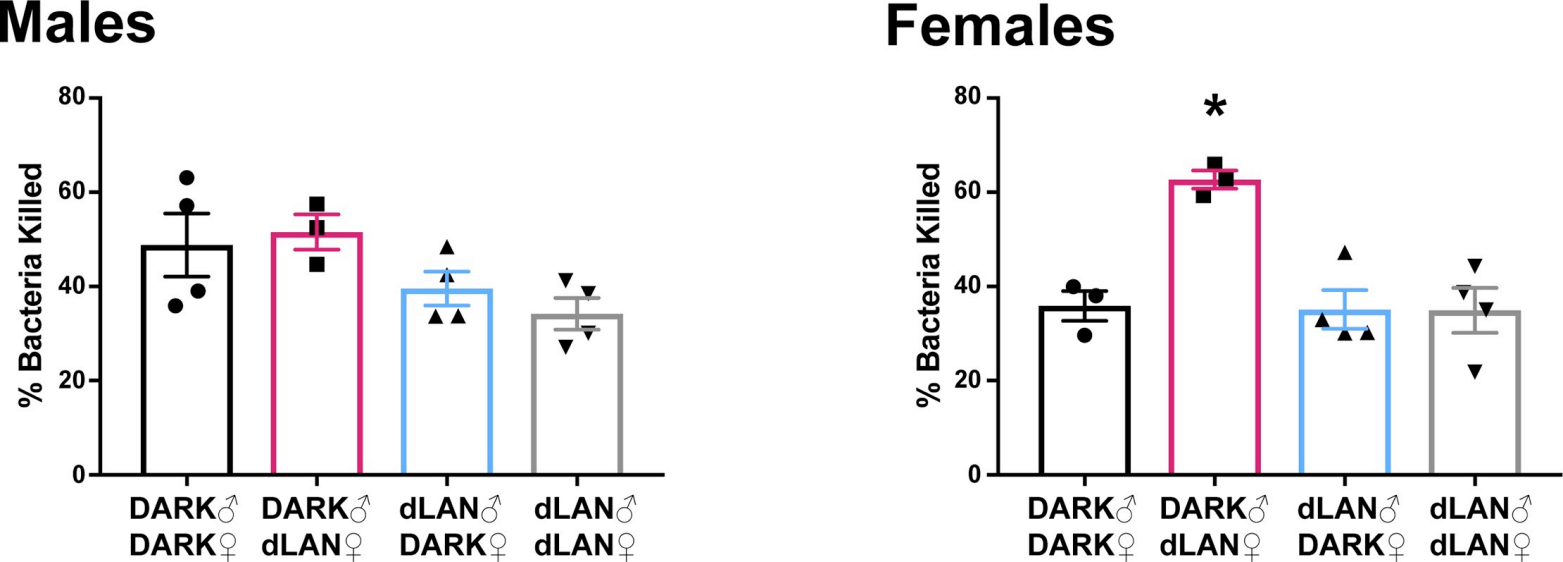
Female offspring of sires exposed to dLAN increased serum bacteria killing capacity. Bacteria killing capacity of serum collected from offspring 3 hours post-LPS challenge. N = 3-4/group/sex; error bars represent SEM. *P < 0.05 DARK-dLAN vs all.

Female, but not male, offspring of dams exposed to dLAN increased serum bactericidal capacity 3 hours post-LPS relative to offspring of DARK-housed pairs (F_3,10_ = 10.67, *p* < 0.01; Fig 5).

#### Preconception exposure to dLAN does not alter maternal care

To rule out indirect effects of dLAN due to changes in maternal care, we examined the average time spent nursing, nursing and licking, or off the nest; maternal care did not differ by parental lighting condition (*p* > 0.05; S1 Fig). Dams previously exposed to dLAN did not differ from DARK-housed dams with regards to fragmentation of maternal care (*p* > 0.05).

## Discussion

Altered immune responses have been documented in adult rodents exposed to dLAN, but the multigenerational effects of nighttime light are only beginning to be appreciated [7–10]. In the current study, we investigated innate immune and febrile responses in the offspring of parents exposed to dLAN *prior* to conception. Maternal exposure to dLAN prior to conception impaired offspring febrile responses and increased splenic *il-1* production in response to LPS, specifically in males, and had an offspring sex-dependent effect on expression of *il-1* in the hypothalamus. Paternal pre-conception dLAN decreased splenic *tnf-α* expression, female offspring splenic *il-6* and hypothalamic *il-1*, and reduced offspring serum bactericidal capacity. These changes occurred despite offspring being conceived, gestated, and reared under standard dark night conditions. Overall, these data suggest that dLAN has multigenerational effects on innate immunity and sickness responses.

Administration of an endotoxin, such as LPS, induces not only peripheral, but central immune responses. Specifically, IL-1 and IL-6 act synergistically in the hypothalamus to induce febrile responses [13]. Febrile temperature was markedly reduced in male DARK-dLAN offspring (Fig 2A) and despite peripheral elevations in splenic *il-1* (Fig 3C), hypothalamic *il-1* was dampened in maternal dLAN male offspring (Fig 4C). However, hypothalamic *il-6* expression in response to LPS are comparable in DARK-dLAN offspring relative to control animals (Fig 4B). The mismatch in peripheral and central *il-1* expression may play a role in altering the febrile response of maternal dLAN male offspring. Of note, these data are representative of whole hypothalamus and may not differentiate cytokine expression in the hypothalamic nuclei responsible for regulating body temperature.

Offspring of sires exposed to dLAN reduced blood bactericidal capacity (Fig 5). Blood bactericidal capacity has been used in humans and animal models to test constitutive innate immunity and predict susceptibility to bacterial infections [14,15]. Serum bactericidal capacity, and specifically killing of *E*.*coli* (ATCC# 0483E7), is dependent on activation of the acute phase response (APR) and complement [14,16]. IL-6 has long been identified as a major regulator of the APR [17]. However, IL-1, IL-6, and TNF have all been reported to play a role in regulating the synthesis of complement components and other acute phase reactants [18–20]. Offspring of sires exposed to dLAN had minimal LPS responses in splenic *tnf-*α and *il-6* gene expression relative to their saline treated counterparts (Fig 3A–3F), especially in females. This relative decrease in cytokine response to LPS did not seem to affect bacteria killing capacity in paternal DARK offspring (Fig 5).

We have previously reported changes in endocrine receptor expression in the spleen of preconception dLAN offspring [10]. Specifically, at the onset of the active phase glucocorticoid receptor (GR) expression decreased in male offspring of dLAN sires to levels comparable to females. Glucocorticoids are typically released in response to an immune challenge to prevent runaway inflammation by acting as a transcription factor for pro- and anti- inflammatory genes [21,22]. Changes in splenic GR expression may alter the fine-tuned transcriptional regulation of the magnitude and duration of immune responses in offspring of parents exposed to dLAN. Although the reported cytokine gene expression only captures a snapshot at the peak of the febrile response, 3 hours post-LPS, offspring of sires exposed to dLAN do reduce *tnf-α* expression relative to offspring of control and dLAN-exposed dams (Fig 3A and 3D). Preconception dLAN offspring also decreased global methylation in the spleen, suggesting lasting effects of preconception exposure to dLAN on the transcriptional landscape of the offspring spleen.

Adult Siberian hamsters exposed to dLAN decrease active phase locomotor activity [23]. Paternal dLAN offspring display a similar decrease in activity during the dark phase (Fig 1A). Homecage locomotor activity serves as an output marker of the circadian system. Decreased locomotor activity during the dark phase may suggest alterations in the circadian system. Indeed, in adult rodents, exposure to dLAN dampens clock gene expression in the SCN and the hippocampus [24,25]. Similar changes in the molecular clock may underlie alterations in other outputs of the circadian system, such as immune regulation.

The modern adoption of electric lighting has occurred without consideration or understanding of the potential for light at night to disrupt circadian organization of physiological, behavioral, or and affective processes. Taken together, our data suggest that chronic pre-conception exposure to dLAN has multigenerational effects on offspring immune, endocrine, and affective responses.

## Supporting information

S1 Checklist (PDF)

S1 Fig Exposure to dLAN prior to conception does not alter maternal care. Percentage of time observed engaged in nursing behavior averaged from three daily observation across the first postnatal week. N = 6-9/group; error bars represent SEM.
(TIF)
