# Supplementary figures and images for: Exposure to dim light at night prior to conception attenuates offspring innate immune responses
Source: PLoS One. 2020 Apr 17;15(4):e0231140. doi: 10.1371/journal.pone.0231140 (PMC7164648; doi:10.1371/journal.pone.0231140)

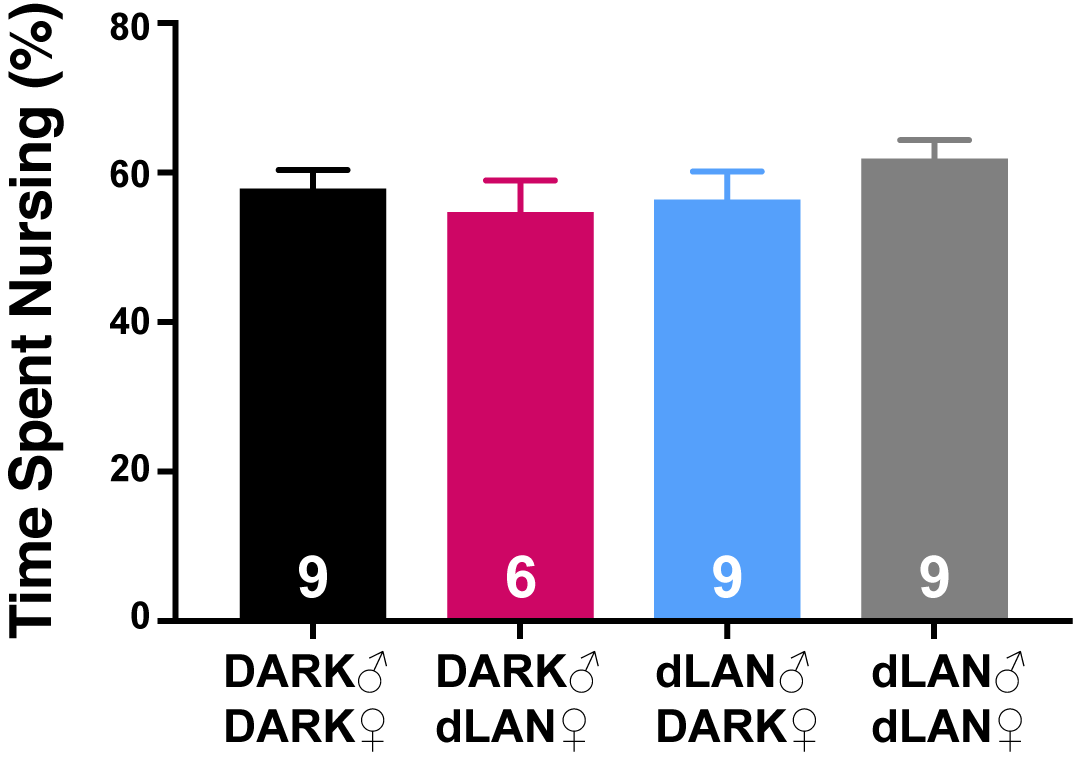

Supplement: S1 Fig — Percentage of time observed engaged in nursing behavior averaged from three daily observation across the first postnatal week. N = 6-9/group; error bars represent SEM. (TIF) [file pone.0231140.s002.tif]
